# Endothelial TLR4 Expression Mediates Vaso-Occlusive Crisis in Sickle Cell Disease

**DOI:** 10.3389/fimmu.2020.613278

**Published:** 2021-01-19

**Authors:** Joan D. Beckman, Fuad Abdullah, Chunsheng Chen, Rachel Kirchner, Dormarie Rivera-Rodriguez, Zachary M. Kiser, Aithanh Nguyen, Ping Zhang, Julia Nguyen, Robert P. Hebbel, John D. Belcher, Gregory M. Vercellotti

**Affiliations:** Department of Medicine, Division of Hematology, Oncology and Transplantation, University of Minnesota, Minneapolis, MN, United States

**Keywords:** sickle cell disease, heme, Toll-like receptor 4, endothelium, vaso-occlusive events

## Abstract

Heme, released from red blood cells in sickle cell disease (SCD), interacts with toll-like receptor 4 (TLR4) to activate NF-κB leading to the production of cytokines and adhesion molecules which promote inflammation, pain, and vaso-occlusion. In SCD, TLR4 inhibition has been shown to modulate heme-induced microvascular stasis and lung injury. We sought to delineate the role of endothelial verses hematopoietic TLR4 in SCD by developing a TLR4 null transgenic sickle mouse. We bred a global *Tlr4^-/-^* deficiency state into Townes-AA mice expressing normal human adult hemoglobin A and Townes-SS mice expressing sickle hemoglobin S. SS-*Tlr4^-/-^* had similar complete blood counts and serum chemistries as SS-*Tlr4*^+/+^ mice. However, SS-*Tlr4^-/-^* mice developed significantly less microvascular stasis in dorsal skin fold chambers than SS-*Tlr4*^+/+^ mice in response to challenges with heme, lipopolysaccharide (LPS), and hypoxia/reoxygenation (H/R). To define a potential mechanism for decreased microvascular stasis in SS-*Tlr4^-/-^* mice, we measured pro-inflammatory NF-κB and adhesion molecules in livers post-heme challenge. Compared to heme-challenged SS-*Tlr4*^+/+^ livers, SS-*Tlr4*^-/-^ livers had lower adhesion molecule and cytokine mRNAs, NF-κB phospho-p65, and adhesion molecule protein expression. Furthermore, lung P-selectin and von Willebrand factor immunostaining was reduced. Next, to establish if endothelial or hematopoietic cell TLR4 signaling is critical to vaso-occlusive physiology, we created chimeric mice by transplanting SS-*Tlr4*^-/-^ or SS-*Tlr4*^+/+^ bone marrow into AA-*Tlr4*^-/-^ or AA-*Tlr4*^+/+^ recipients. Hemin-stimulated microvascular stasis was significantly decreased when the recipient was AA-*Tlr4^-/-^*. These data demonstrate that endothelial, but not hematopoietic, TLR4 expression is necessary to initiate vaso-occlusive physiology in SS mice.

## Introduction

Sickle cell disease (SCD), which is caused by a single point mutation in the β-globin gene of hemoglobin, manifests with chronic intra- and extravascular hemolysis, oxidative stress, inflammation, and vaso-occlusive crisis (VOC). Recently, the role of the innate immune system in perpetuating SCD inflammation and vaso-occlusive physiology has been recognized (1–9). Specifically, heme, which is released during intravascular hemolysis, is able to serve as a damage-associated molecular pattern (DAMP) to stimulate TLR4 signaling on blood cells and the vasculature leading to vaso-occlusion and pulmonary injury (8–11). Heme mediates pain *via* TLR4 in SCD mice and blockade or knockout of TLR4 attenuates hyperalgesia suggesting heme -induced microglial activation *via* TLR4 in the central nervous system contributes to the initiation and maintenance of sickle pain (12). Consequentially, downstream of TLR4, activation of the pro-inflammatory transcription factor NF-κB leads to the production of cytokines and adhesion molecules that promote inflammation, coagulation, and vaso-occlusion (8, 9). Additionally, work done in drug-induced hemolysis models suggests that TLR4-mediated P-selectin release increases complement activation to further drive endothelial activation (13). Collectively, these studies have raised speculation that TLR4 and complement-targeted therapies may reduce severity of VOC in SCD.

Several critical questions regarding the consequences of TLR4 inhibition in SCD remain. Our prior work demonstrated that knockout of TLR4 in the vessel wall was sufficient to ablate SCD VOC physiology (8). However, those transplant studies of SS bone marrow into TLR4 knockout mice could not examine the effects of TLR4 knockout in hematopoietic cells on VOC. We previously showed that monocytes isolated from SCD patients can activate endothelial monolayers and others have shown that heterocellular aggregates play an important role in vaso-occlusion (14–16). Here we asked the question, does knockout of TLR4 in circulating hematopoietic-derived cells, but not the vessel wall, ablate microvascular stasis?

Therefore, we bred a global *Tlr4^-/-^* deficiency state into Townes-AA mice expressing normal human adult hemoglobin A and Townes-SS mice expressing sickle hemoglobin S. We demonstrate that loss of TLR4 in SCD does not alter chronic hemolysis, but does decrease response to an acute stimulus with hemin, LPS or ischemia through loss of downstream NF-κB signaling. Downstream of NF-κB, SS-*Tlr4^-/-^* mice exhibit decreased pro-inflammatory and adhesive protein expression. Importantly, using bone marrow chimeras, we demonstrated that endothelial, but not hematopoietic, TLR4 signaling is critical in mediating SCD VOC.

## Materials and Methods

### Mice

All animal experiments were approved by the University of Minnesota’s Institutional Animal Care and Use Committee. These studies used male and female Townes-AA and -SS mice on a 129/B6 mixed genetic background (17) and *Tlr4*^-/-^ mice (TLR4^lps-del,^ Jackson Labs) with knockout of the entire *Tlr4* gene, expressing murine alpha and beta globins on a C57B6 genetic background. We bred a global *Tlr4^-/-^* deficiency state into Townes-AA mice expressing normal human adult hemoglobin A and Townes-SS mice expressing sickle hemoglobin S. These *Tlr4*^-/-^ Townes mice were backcrossed 10 generations to homogenize their genetic background with our *Tlr4^+/+^* Townes mouse colony. All animals were housed in specific pathogen-free rooms to limit infections and kept on a 12 hour (h) light/dark cycle at 21°C. All animals were monitored daily for health problems, food and water levels, and cage conditions. All animals were included in each endpoint analysis and there were no unexpected adverse events that required modification of the protocol. Mice were aged 8–24 weeks.

### Bone Marrow Transplants

Chimeric mice were generated by harvesting bone marrow (BM) from SS-*Tlr4*^+/+^ or SS-*Tlr4*^-/-^ mice followed by transplant into lethally irradiated AA-*Tlr4^+/+^* or AA-*Tlr4*^-/-^ mice. Recipients (8–10 weeks of age) were irradiated with 2 doses of 5 Gy (X-RAD 320 Biological Irradiator) 3 hours apart. During the 3-hour interval, BM donors were sacrificed and BM was collected from both femurs. Ten million BM cells were injected *via* tail vein into each irradiated recipient. Drinking water containing 0.2% neomycin sulfate (Sigma-Aldrich) was given to transplanted mice for 3 weeks immediately after transplantation. Eight weeks post-transplant, globin phenotype was confirmed by hemoglobin isoelectric focusing and *Tlr4* genotype was verified by PCR. Chimeric mice were employed 16 to 24 weeks after transplant.

### Blood Analysis

Blood was collected *via* cardiac puncture at the time of euthanasia from mice into sodium EDTA or serum separator tubes at time points indicated. Complete blood counts with differential, hematocrit levels, and reticulocytes were measured in EDTA blood by the University of Minnesota Veterinary Diagnostic Laboratory.

### Measurement of Vaso-Occlusion (Microvascular Stasis)

Mice were anesthetized with a mixture of ketamine (106 mg/kg) and xylazine (7.2 mg/kg) and implanted with dorsal skin-fold chambers (**Supplemental Figure 1**). After implantation, mice were placed on an intravital microscopy stage and 20–24 flowing subcutaneous venules in the chamber window were selected and mapped as previously described (18). After baseline selection of flowing venules, mice were infused with a bolus infusion *via* tail vein with the indicated doses of hemin (3.2 µmol/kg) or lipopolysaccharide (LPS, 1 mg/kg; Escherichia coli, serotype O111:B4; Sigma-Aldrich) or exposed to H/R which consisted of 1-hour hypoxia (7%O_2_/93%N_2_) followed by 4-hours normoxia. Each of the same venules selected and mapped at baseline were visually re-examined for stasis (no flow) at 1, 2, 3, and 4 hours after infusion or H/R. The static venules in each mouse were counted and percent stasis at 1–4 h was calculated by dividing the number of static venules by the total (static + flowing) number of venules.

### Western Blots

Microsomes and nuclear extracts were isolated from tissues of mice as previously described (19). Immunoblots of cellular subfractions (15–30 μg of protein) were immunostained with primary antibodies to NF-ĸB phospho-p65 (Ser536, Cell Signaling #3031), total p65 (Cell Signaling #3034), VCAM-1 (Abcam #174279), ICAM-1 (Abcam #ab124759), E-selectin (BioVision #3631) and loading control GAPDH (Sigma-Aldrich #G9545). Primary antibodies were detected with appropriate secondary antibodies conjugated to alkaline phosphatase and visualized with ECF substrate (GE Healthcare) and a Typhoon FLA 9500 imager (GE Healthcare).

### RNA Analysis

RNA was extracted using RNeasy kit (Qiagen), followed by cDNA generation according to the manufacturer’s protocol (Bio-Rad). Prime PCR RNA array was used for genes. Each reaction contained 20 ng of cDNA, lyophilized primers, SSoAdvanced SYBR Green QPCR master mix (BioRad). The PCR conditions included activating the DNA polymerase at 95°C for 10 min, followed by 40 cycles of three step PCR (95°C for 10 s, 60°C for 30 s). Melt curves for each primer set was run and verified. The cycle threshold (*Ct*) values from samples of each gene and the internal control (GAPDH) were obtained and the relative quantification for each gene was calculated using the ΔΔ*Ct* method (20).

### Immunohistology

Mice were infused with hemin (3.2 μmol/kg) 4 hours before tissue collection. Lungs were collected and placed in optimal cutting temperature (OCT) compound, snap-frozen in liquid nitrogen and stored at -85°C prior to frozen sectioning in a microtome-cryostat into 6 µm sections. Tissues were stained with primary antibodies to P-selectin (R&D Systems #AF737) and von Willebrand factor (vWF, Cedarlane #CL20176A-R), and with the nuclear stain DAPI (Sigma-Aldrich). Primary antibodies in tissues were identified with the appropriate fluorescent-labeled secondary antibodies (Jackson Immunoresearch). Slides were mounted using DPX mounting medium (Electron Microscope Sciences #13514), visualized, and images acquired using a FluoView FV1000 BX2 upright confocal microscope (Olympus, Center Valley, PA) with UPlanSApo 20X/0.80 and UPlanApo N 60X/1.42 objectives with zoom (Z) 2. Images were processed with FluoView (Olympus) and Adobe Photoshop software (San Jose, CA).

### Statistics

Descriptive statistics are presented as mean ± standard error. Normality assessments were conducted for groups. Analysis for each experiment is included in legends, with multiple comparisons analyzed using ANOVA with the Holm-Sidak method or Kruskal-Wallis with the Dunn’s test for multiple comparisons using GraphPad Prism (v 8).

## Results

### Generating Townes-AA and –SS *Tlr4* Knockout Mice

In SCD mice, inhibition of TLR4 signaling using the small molecule inhibitor TAK-242 reduces microvascular stasis in presence of hemin, LPS, and hypoxia/reoxygenation (H/R) (8, 9). Furthermore, TLR4 inhibition prevents hemin-mediated lethality. Therefore, to determine if knockout of *Tlr4* in mice carrying human hemoglobin S (Townes-SS) would reduce hemolysis and inflammation, we generated Townes-SS mice with *Tlr4*^-/-^ genotype. *Tlr4*^-/-^ mice (TLR4^lps-del,^ Jackson Labs) with knockout of the entire *Tlr4* gene, expressing murine alpha and beta globins on a C57B6 genetic background were breed with Townes-AA *Tlr4*^+/+^ mice expressing human alpha- and beta-globins on a mixed 129/B6 genetic background. Male and female heterozygous offspring were bred together and pups expressing exclusively human alpha- and beta-globins and at least one deleted *Tlr*4 gene were selected for backcrossing 9 generations with Townes-AA *Tlr4*^+/+^ mice from our colony with AA mice heterozygous for *Tlr*4 knockout selected for breeding with AA-*Tlr4*^+/+^ mice at each new generation. After 9 backcrosses, heterozygous AA-*Tlr4*^+/-^ were bred with SS-*Tlr4*^+/+^ mice from the colony for the 10^th^ backcross. AS-*Tlr4*^+/-^ offspring were breed together and AA, AS and SS-Tlr4^-/-^ offspring were selected for breeding to expand the Townes-AA-, AS-, and SS-*Tlr4*^-/-^ colony and generate mice for experimentation (**Supplemental Figure 2**).

Compared to SS-*Tlr4*^+/+^ mice, SS-*Tlr4*^-/-^ mice had no differences in white blood cell counts or in markers of hemolysis (**Table 1**). Likewise, there was no difference in organ function, as demonstrated by serum chemistries (**Table 1**). Therefore, knockout of the *Tlr4* gene in SS mice does not appear to reduce chronic hemolysis.

**Table 1:** Complete blood count and serum chemistries from AA-*Tlr4*^+/+^, AA-*Tlr4*^-/-^, SS-*Tlr4*^+/+^ and SS-*Tlr4*^-/-^ mice.

Complete Blood Count	AA-*Tlr4*^+/+^ (n = 4–5, ± sth dev)	AA-*Tlr4*^-/-^ (n = 5–6, ± sth dev)	SS-*Tlr4*^+/+^ (n = 5–6, ± sth dev)	SS-*Tlr4*^-/-^ (n = 4, ± sth dev)
**White blood cells**	1.68 ± 0.68	2.04 ± 1.20	36.62 ± 19.0	33.24 ± 9.66
**(WBC), 10^3^/µl**				
** Neutrophils (%)** ** Lymphocytes (%)** ** Monocytes (%)** ** Eosinophils (%)** ** Basophils (%)**	22.8 72.8 1.3 2.5 0.8	19.4 76.6 1.8 1.8 0.4	9.8 87.3 2.5 0.0 0.3	14.5 80.8 3.3 0.8 0.8
**Red blood cells** **(RBC), 10^6^/µL**	10.8 ± 1.1	11.3 ± 1.8	5.1 ± 1.1	4.4 ± 1.1
**Hemoglobin (Hgb). g/dL**	9.5 ± 0.9	9.8 ± 1.5	4.8 ± 1.0	3.9 ± 1.1
**Hematocrit (Hct), %**	32.1 ± 5.6	35.2 ± 6.1	20.6 ± 4.8	20.3 ± 3.0
**Platelets, 10^3^/µl**	700 ± 112	1012 ± 74	480.1 ± 65.3	445.5 ± 63.3
**Reticulocytes (%)**	6.6 ± 1.4	11.8 ± 7.9	40.2 ± 29.1	44.9 ± 26.9
**Serum Chemistries**	**AA-*Tlr4*^+/+^**	**AA-*Tlr4*^-/-^**	**SS-*Tlr4*^+/+^**	**SS-*Tlr4*^-/-^**
**AST**	169 ± 43.5	132.7 ± 45.8	653.4 ± 422	416.5 ± 299
**ALT**	73.2 ± 22.6	57.2 ± 25.7	515.8 ± 478	617.3 ± 720
**Total bilirubin**	0.1 ± 0.05	0.2 ± 0.11	1.5 ± 0.80	1.2 ± 0.25
**Albumin**	2.5 ± 0.14	2.5 ± 0.23	2.9 ± 0.13	2.6 ± 0.23
**Blood urea nitrogen**	26.4 ± 4.3	22.8 ± 4.4	22.0 ± 2.9	25.0 ± 3.2
**Creatinine**	0.2 ± 0.08	0.1 ± 0.09	0.1 ± 0.1	0.2 ± 0.08

### *Tlr4* Knockout Reduced Microvascular Stasis in Sickle Cell Mice

**Figure 1:**
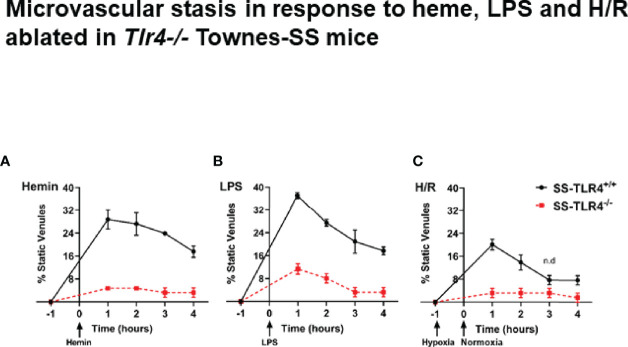
Townes SS-*Tlr4*^-/-^ mice are protected from developing microvascular stasis under inflammatory stimuli. **(A)** Microvascular stasis in Townes SS-*Tlr4*^+/+^ (black) and SS-*Tlr4*^-/-^ (red) mice after stimulation with 3.2 µmol/kg hemin. **(B)** Microvascular stasis after stimulation with LPS (1 mg/kg). **(C)** Microvascular stasis after 1 h hypoxia at 7%. All treatment groups with n = 4 mice/group. P < 0.05 for all time point except where n.d. is present to signify no difference as done by multiple t-tests *via* Holm-Sidak method.

**Figure 2:**
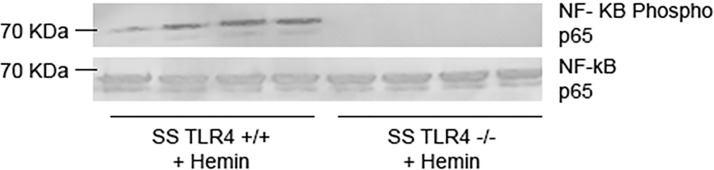
Hemin-stimulated Townes SS-*Tlr4*^-/-^ mice do not activate NF-κβ signaling. Western blot of liver nuclear extracts isolated from hemin-stimulated Townes SS-*Tlr4*^+/+^ and SS-*Tlr4*^-/-^ mice probed for NF-κB phopsho-p65 and total p65. (n = 4 per group).

We have previously demonstrated that compared to AA mice, SS mice exhibit a chronic baseline hemolysis that leads to increased occlusion of skin venules (8). However, SS, but not AA mice, also exhibit robust microvascular vaso-occlusion when stimulated with excess hemin, LPS, or H/R (8). Therefore, to determine if TLR4 knockout would protect SS mice from vaso-occlusion, we used dorsal skin fold chambers to assess microvascular stasis at 1h, 2h, 3h, and 4h post-stimulation with hemin, LPS or H/R in SS-*Tlr4*^+/+^ and SS-*Tlr4*^-/-^ mice (**Supplemental Figure 1**). Compared to hemin-stimulated SS-*Tlr4*^+/+^ mice, hemin-stimulated SS-*Tlr4*^-/-^ mice had a significant reduction in % venules occluded at 1–4 h post-infusion (*Tlr4*^+/+^ % occluded range 17.5%-28.7% versus *Tlr4*^-/-^ % occluded range 1.8%-3.9%, p < 0.005, **Figure 1A**). With LPS stimulation, compared the SS-*Tlr4*^+/+^ mice, SS-*Tlr4*^-/-^ mice also exhibited decreased % venules occluded at 1–4 h post-infusion (*Tlr4*^+/+^ % occluded range 17.7–37.1% vs. *Tlr4*^-/-^ % occluded range 3.3–11.3%, p < 0.02, **Figure 1B**). After H/R, compared to SS-*Tlr4*^+/+^, SS-*Tlr4*^-/-^ exhibited decreased occlusion at 1 h (*Tlr4*^+/+^ % occluded 20.1 vs. *Tlr4*^-/-^ % occluded 3.2%, p < 0.01, **Figure 1C**) and 2 h time points (*Tlr4*^+/+^ % occluded 13.9 vs. *Tlr4*^-/-^ % occluded 3.2%, p < 0.01, **Figure 1C**). Collectively, these data suggest that loss of TLR4 in SS mice does not reduce baseline hemolysis, but does eliminate microvascular stasis after challenge with hemin, LPS, or H/R.

### Loss of TLR4 Reduces NF-ĸB Signaling

The innate immune system activates signaling cascades within cells in order to promote inflammation. The TLR4 and NADPH oxidase (NOX)-dependent signaling cascades converge to increase pro-inflammatory NF-κB signaling (21). When stimulated with hemin, *Tlr4^-/-^* mouse pulmonary vein endothelial cells demonstrate reduced NF-κB activation (8). Likewise, treatment of human umbilical vein endothelial cells with the TLR4 inhibitor TAK-242 also reduces NF-κB signaling. To evaluate if knockout of TLR4 reduces NF-κB signaling in SS mice, we performed western blots on nuclear extracts from livers isolated from hemin-stimulated SS-*Tlr4*^+/+^ and SS-*Tlr4*^-/-^ mice. Compared to hemin-stimulated SS-*Tlr4*^+/+^, hemin-stimulated SS-*Tlr4*^-/-^ mice lacked phosphorylation of NF-κB p65 (**Figure 2**). This suggests that loss of TLR4 reduces inflammation through abrogation of NF-κB signaling.

### SS-*Tlr4*^-/-^ Mice Challenged With Hemin Exhibit Reduced Pro-Inflammatory Cytokine and Adhesion Molecule mRNA

**Figure 3:**
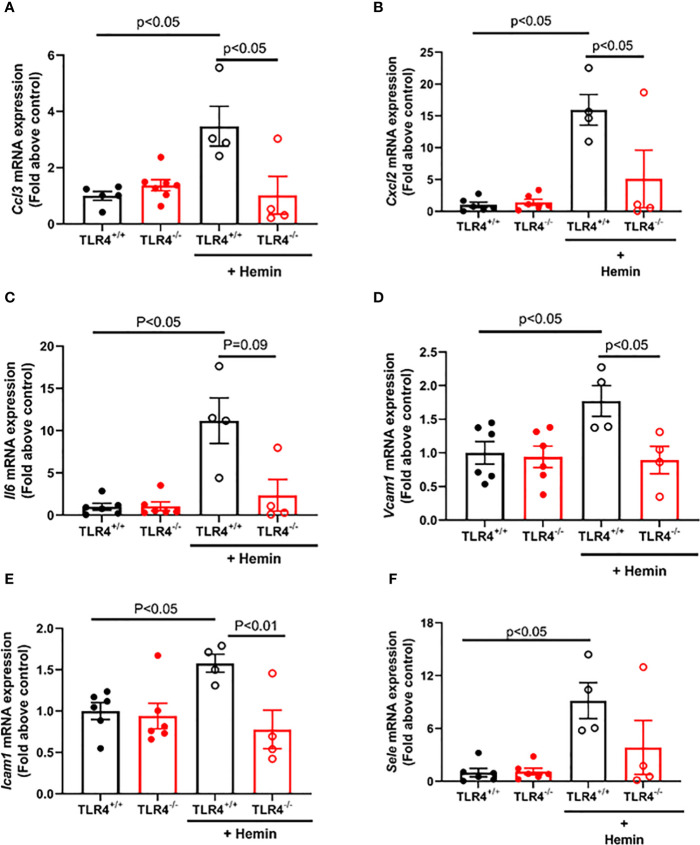
Hemin-stimulated Townes SS-*Tlr4*^-/-^ mice do not upregulate pro-inflammatory cytokine and adhesive genes. Liver mRNA was extracted and underwent qRT-PCR analysis. **(A)** Macrophage inflammatory protein 1-α (MIP-1α, *Ccl3*). **(B)** Macrophage inflammatory 2-α (MIP-2α, *Cxcl2*) mRNA. **(C)** IL-6 *(Il6)* mRNA. **(D)** *Vcam1* mRNA **(E)** *Icam1* mRNA. **(F)** E-selectin (*Sele*) mRNA. Values are mean ± standard error of mean (SEM), with p-values determined by one-way analysis of variance with Holm-Sidak’s multiple comparison testing.

Vaso-occlusion requires both inflammation and adhesion to occur, with NF-κB signaling serving as a crucial transcription signaling for numerous pro-inflammatory and adhesive genes. Therefore, we sought to evaluate the downstream consequences of reduced NF-κB activation in hemin-stimulated SS-*Tlr4*^-/-^ mice by assessing the livers of SS-*Tlr4*^+/+^ and SS-*Tlr4*^-/-^ mice for changes in pro-inflammatory and adhesion gene expression. First, compared to untreated SS-*Tlr4*^+/+^ mice, hemin-treated SS-*Tlr4*^+/+^ mice have significant upregulation of macrophage inflammatory protein 1-α (MIP-1α, *Ccl3*) and macrophage inflammatory 2-α (MIP-2α, *Cxcl2*) mRNA by 3.5-fold (*Ccl3*, P < 0.004, n = 4–5 mice per group, **Figure 3A**) and 16-fold respectively (*Cxcl2*, P < 0.0004, n = 4–5 mice per group, **Figure 3B**). Compared to untreated SS-*Tlr4*^-/-^ mice, hemin-stimulated SS-*Tlr4*^-/-^ mice do not upregulate either *Ccl3* mRNA (**Figure 3A**) or *Cxcl2* mRNA (**Figure 3B**). Moreover, for both chemokines, comparison between hemin-treated SS-*Tlr4*^+/+^ mice to hemin-treated SS-*Tlr4*^-/-^ mice demonstrated significant loss of *Ccl3* and *Cxcl2* mRNA upregulation in absence of TLR4. Next, we assessed IL-6 (Il6) mRNA expression. Similar to *Ccl3* and *Cxcl2*, compared to untreated SS-*Tlr4*^+/+^ mice, hemin-treated SS-*Tlr4*^+/+^ mice had an 11-fold increase in *Il6* mRNA (P < 0.03, n = 4–6 mice per group, **Figure 3C**). In untreated and hemin-stimulated SS-*Tlr4*^-/-^, there was no difference in *Il6* mRNA (n = 4–6 mice per group, **Figure 3C**). Additionally, comparison between hemin-treated SS-*Tlr4*^+/+^ mice to hemin-treated SS-*Tlr4*^-/-^ mice demonstrated a reduced *Il6* mRNA upregulation (p=0.09) in absence of TLR4. This was also seen in the kidneys (**Supplemental Figure 3A**). Collectively, these data suggest that loss of TLR4 in SCD leads to decreased inflammation-mediated cytokine gene expression.

Next, to determine if loss of TLR4 reduces hemin-mediated upregulation of endothelial adhesion molecules, we assessed mRNA expression of *Vcam1*, *Icam1*, and E-selectin (*Sele*). Compared to untreated SS-*Tlr4*^+/+^ mice, hemin-stimulated SS-*Tlr4*^+/+^ mice had a significant 1.8-fold upregulation of *Vcam1* mRNA (P < 0.05, **Figure 3D**) and 1.6-fold upregulation of *Icam1* mRNA (P < 0.05, **Figure 3E**). In SS-*Tlr4*^-/-^ mice, compared to untreated, hemin-stimulated SS-*Tlr4*^-/-^ mice had no difference in *Vcam1* mRNA (**Figure 3D**) or *Icam1* mRNA (**Figure 3E**). Similar to pro-inflammatory markers, comparison between hemin-treated SS-*Tlr4*^+/+^ mice and SS-*Tlr4*^-/-^ mice demonstrated significant loss of *Vcam1* mRNA and *Icam1* mRNA upregulation in absence of TLR4. Last, for *Sele*, compared to untreated SS-*Tlr4*^+/+^ mice, hemin-stimulated SS-*Tlr4*^+/+^ mice upregulated *Sele* mRNA 9-fold (P < 0.05, **Figure 3F**), whereas compared to untreated SS-*Tlr4*^-/-^mice, hemin-stimulated SS-*Tlr4*^-/-^mice did not upregulate *Sele* mRNA (**Figure 3F**). Similar pattern of mRNA expression changes were also observed for these genes in the kidney (**Supplemental Figures 3B–D**). Collectively, these data suggest that in SS mice, loss of TLR4 reduces upregulation of both pro-inflammatory and endothelial adhesion genes.

### Hemin-Stimulated SS-*Tlr4*^-/-^ Mice Do Not Upregulate Endothelial Adhesion Proteins

**Figure 4:**
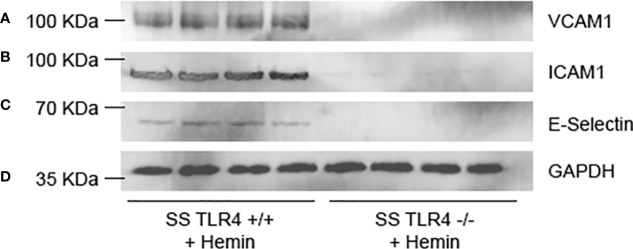
Hemin-stimulated Townes SS-*Tlr4*^-/-^ mice lose upregulation of adhesion proteins. Western blot of liver microsomes isolated from hemin-stimulated Townes SS-*Tlr4*^+/+^ and SS-*Tlr4*^-/-^ mice probed for **(A)** VCAM-1, **(B)** ICAM-1, **(C)** E-selectin, and **(D)** GAPDH loading control. (n = 4 per group).

In SCD, upregulation of endothelial adhesion proteins contributes to vaso-occlusion. Therefore, to evaluate if endothelial adhesion molecule protein expression is reduced in SS mice by TLR4 knockout, we performed western blots on liver microsomes isolated from SS-*Tlr4*^+/+^ and SS-*Tlr4*^-/-^ mice after hemin stimulation. Consistent with mRNA data, compared to hemin-stimulated SS-*Tlr4*^+/+^ mice, hemin-stimulated SS-*Tlr4*^-/-^ mice do not increase VCAM-1 (**Figure 4A**). Likewise, compared to hemin-stimulated SS-*Tlr4*^+/+^ mice, hemin-stimulated SS-*Tlr4*^-/-^ mice do not increase ICAM-1 or E-selectin (**Figures 4B–D**). Together with mRNA data, these data suggest that loss of TLR4 in SS mice reduces heme-mediated endothelial cell activation leading to reduced adhesion molecule expression and decreased inflammation.

### Hemin-Stimulated SS-*Tlr4*^-/-^ Mice Exhibit Reduced P-Selectin and VWF Release From Endothelium

**Figure 5:**
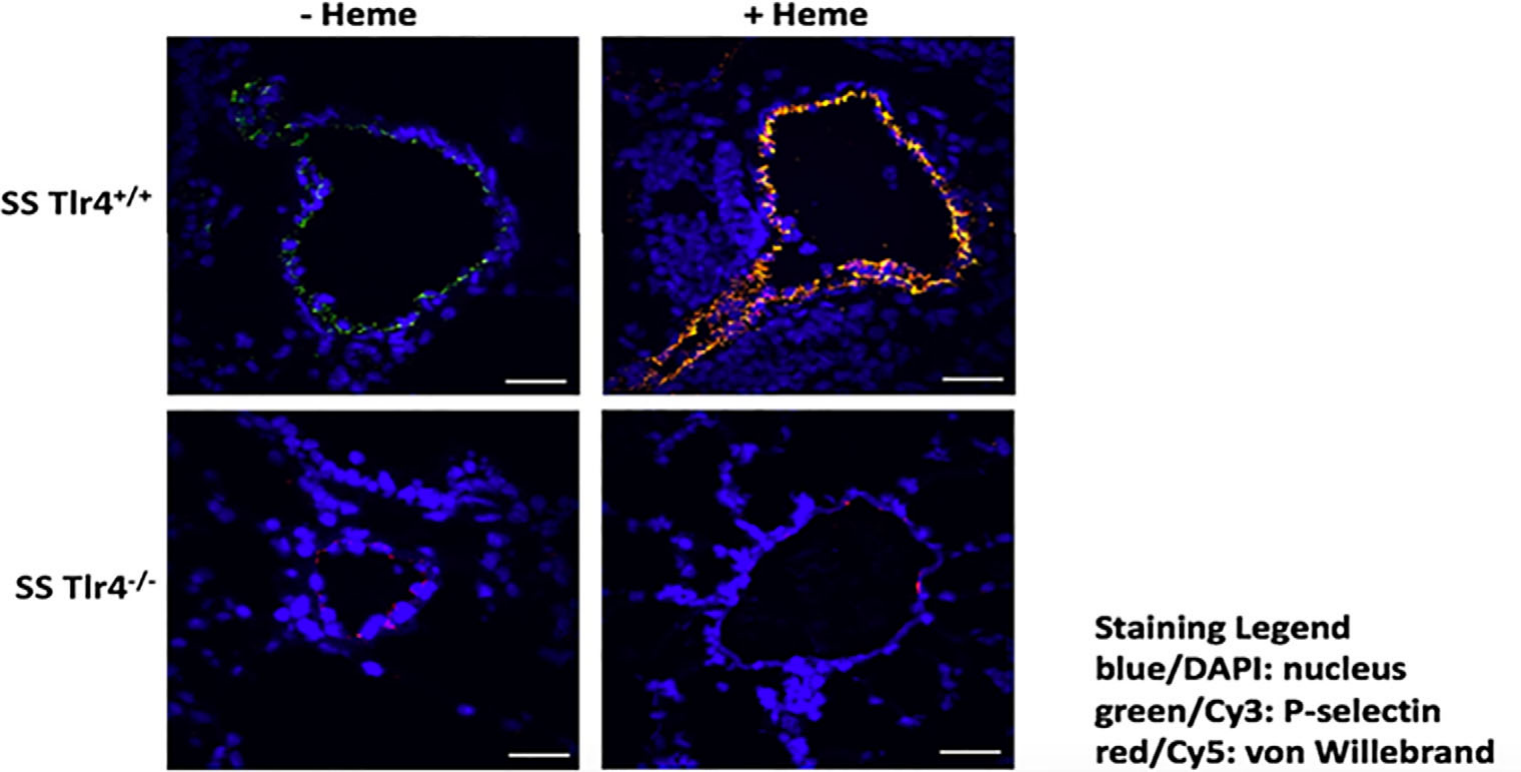
Hemin-stimulated Townes SS-*Tlr4*^-/-^ mice have decreased pulmonary expression of VWF and P-selectin. Immunostaining of surface P-selectin (green) and von Willebrand factor (red) on blood vessels in the lungs of hemin-infused Townes-SS *Tlr4^+/+^* and *Tlr4^-/-^* mice (3.2 µmol/kg and lungs removed at 1h). Scale bars (white) 30 µm. Representative images are presented.

TLR4 blockade reduces heme-mediated release of P-selectin and VWF from endothelial cell Weibel Palade bodies (8). Therefore, we performed immunofluorescence of SS-*Tlr4*^-/-^ and SS-*Tlr4*^+/+^ lungs in mice treated with and without hemin to evaluate heme-mediated P-selectin and VWF release. Compared to SS-*Tlr4*^+/+^mice, SS-*Tlr4*^-/-^ treated with hemin exhibit decreased P-selectin and VWF release (**Figure 5**). Collectively, these data confirm that loss of TLR4 signaling in SS mice reduces pro-adhesive and thrombotic P-selectin and VWF secretion from endothelial Weibel-Palade bodies.

### Endothelial, Not Hematopoietic, TLR4 Drives SS Vaso-Occlusion

**Figure 6:**
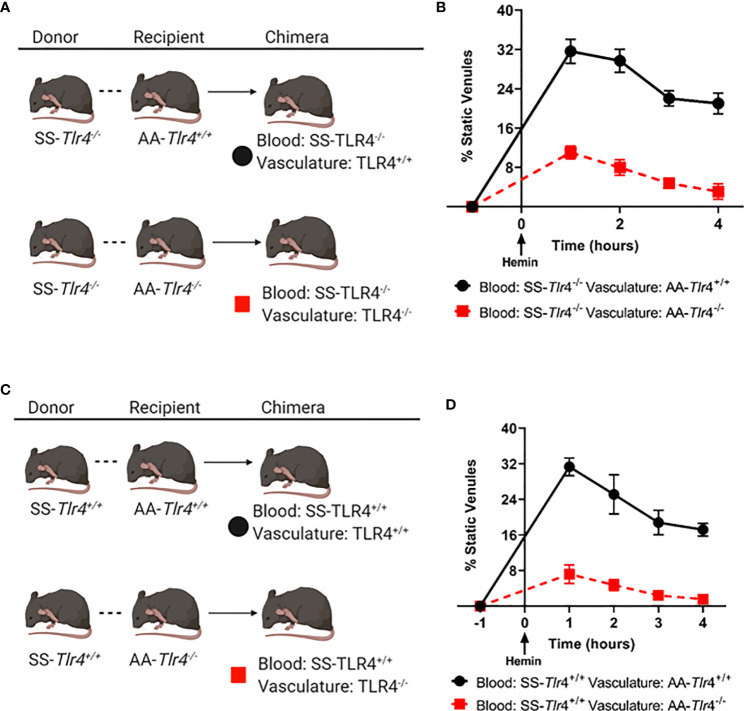
Loss of endothelial, but not hematopoietic, TLR4 expression prevents microvascular stasis. **(A)** Schematic for bone marrow chimera crosses using SS-*Tlr4^-/-^* donor marrow into AA-*Tlr4^+/+^* or AA-*Tlr4^-/-^* recipients. **(B)** Hemin-stimulated microvascular stasis in chimeric AA-*Tlr4*^+/+^ (black) and AA-*Tlr4*^-/-^ (red) recipients that received SS-*Tlr4*^-/-^ marrow. **(C)** schematic for bone marrow chimera crosses using SS-*Tlr4^+/+^* donor marrow into AA-*Tlr4^+/+^* or AA-*Tlr4^-/-^* recipients. **(D)** Hemin-stimulated microvascular stasis in chimeric AA-*Tlr4*^+/+^ (black) and AA-*Tlr4*^-/-^ (red) that received SS-*Tlr4*^+/+^ marrow. All treatment groups with n = 4 mice/group. P < 0.05 for all time points analyzed by multiple t-tests *via* Holm-Sidak method.

Several groups have demonstrated that knockdown of TLR4 in endothelial cells reduces monocyte and neutrophil adhesion (22, 23). Further, prior work demonstrated that knockout of TLR4 in the vessel wall was sufficient to ablate SCD VOC physiology (1). However, those studies transplanted SS BM into TLR4 knockout mice and therefore could not examine the effects of TLR4 knockout in circulating hematopoietic cells on VOC. This is an important question because monocytes isolated from SCD patients can activate endothelial monolayers (14) and heterocellular aggregates play an important role in vaso-occlusion (15, 16). Here we addressed the question does knockout of TLR4 in circulating hematopoietic-derived cells ablate microvascular stasis? Therefore, we performed BM chimera studies using the SS-*Tlr4*^-/-^ and SS-*Tlr4*^+/+^ mice into AA-*Tlr4*^-/-^ or AA *Tlr4*^+/+^ recipients followed by assessment of hemin-stimulated vaso-occlusion in dorsal skin fold chambers (**Figures 6A, C**). First, to assess the contribution of hematopoietic expression of TLR4, we compared chimeras generated from SS-*Tlr4*^-/-^ mice transplanted into AA-*Tlr4*^+/+^ or AA-*Tlr4*^-/-^ recipients (**Figure 6A**). Hemin-stimulated SS or SS-*Tlr4*^+/+^ mice exhibit average % occlusion of ~30% (historic and **Figure 1A**); loss of TLR4 in hematopoietic cells (SS-*Tlr4*^-/-^ mice into AA-*Tlr4*^+/+^ recipients) lead to no change in % venules occluded (range % occluded 21.0–31.6%, **Figure 6B**). Comparatively, and consistent with SS-*Tlr4*^-/-^ mice, transplant of SS-*Tlr4*^-/-^ marrow into AA-*Tlr4*^-/-^ recipients significantly reduced the % venules occluded (range 3.1–11.0% occluded vessels, p < 0.001, **Figure 6B**). Overall, these data suggest that hematopoietic TLR4 signaling is not essential in triggering vaso-occlusive response. Next, to assess effects of endothelial TLR4 knockout, we created chimeras transplanting SS-*Tlr4*^+/+^ hematopoietic cells into AA-*Tlr4*^+/+^ and AA-*Tlr4*^-/-^ recipients (**Figure 6C**). Similar to SS and SS-*Tlr4*^+/+^ mice, transplant of SS-*Tlr4*^+/+^ hematopoietic cells into AA-*Tlr4*^+/+^ lead to vaso-occlusion (range 17.2–31.3% occluded vessels **Figure 6D**). Strikingly, transplant of SS-*Tlr4*^+/+^ marrow into AA-*Tlr4*^-/-^ mice abrogated vessel occlusion (range 1.6–7.2% occluded vessels, p < 0.001, **Figure 6D**). Collectively, these data demonstrate that endothelial, but not hematopoietic, TLR4 expression is necessary to initiate vaso-occlusive physiology in SS mice.

## Discussion

Intravascular hemolysis of sickle red blood cells release hemoglobin S (HbS) into the plasma which is promptly oxidized to methemoglobin, which readily releases free heme. Heme is a DAMP that can activate the innate immune pattern recognition receptor complex of CD14, MD-2 and TLR4 (8–10); this process promotes a pro-inflammatory and pro-adhesive phenotype, which ultimately leads to VOC (1, 8–10). Herein, we demonstrate that loss of TLR4 signaling in SCD leads to decreased VOC stimulated by numerous agonists, including heme, LPS and H/R. Importantly, we also demonstrate that endothelial, but not hematopoietic, TLR4 expression is necessary to initiate vaso-occlusion in SS mice. Collectively, these data illustrate the indispensable role of the endothelium in mediating the crosstalk between hemolysis and the innate immune system in SCD VOC physiology.

In SCD, chronic hemolysis leads to organ dysfunction due to recurrent cycles of I/R physiology. However, VOC occurs when pro-inflammatory stimuli are present, such as heme or LPS and the compensatory mechanisms responsible for rebalancing the system are overcome (24). Therefore, when approaching SCD pathogenesis, acute verses chronic stimulation of the innate immune system should be considered. Our work demonstrates that compared to Townes SS-*Tlr4*^+/+^ mice, Townes SS-*Tlr4*^-/-^ mice did not have changes in baseline hemolytic markers. However, importantly, our studies demonstrate that in response to an acute increase in heme, such as would be expected during an acute VOC, loss of TLR4 signaling results in decreased pro-inflammatory and adhesive gene expression and ultimately decreased stasis. Therefore, we speculate that during VOC, strategies to target TLR4 may reduce incidence and perhaps duration of VOC.

In SCD, the prominent end-organ damage is associated with the vasculature, including pulmonary hypertension, strokes, and priapism and retinal disease. However, renal failure, liver damage and hyposplenism are also manifestations of chronic, progressive I/R damage (25). Therefore, we performed analysis of inflammatory markers and adhesion markers in the liver, lungs and kidneys. In the liver, resident hepatic macrophages, also known as Kupffer cells, and hepatic stellate cells are key mediators of hepatic fibrogenesis. Hepatic stellate cells are the main target of TLR4 ligands in the liver (26). Once stimulated, hepatic stellate cells stimulate chemokine secretion, which drives Kupffer cell chemotaxis and pro-fibrotic TGF-β production. Overall, it has been found that loss of hepatic stellate cell TLR4-MyD88 signaling reduces development of hepatic fibrosis. Of note, during chimera generation, hepatic stellate cells are not replaced by BM-derived cells, and without clodraonate-mediated depletion, only a proportion of Kupffer cells become replaced by BM-derived cells (26, 27). Therefore, in our chimera studies, the AA-*Tlr4^-/-^* recipients lack hepatic stellate cells responsive to TLR4 ligands. Of note, when comparing SS-*Tlr4^-/-^* mice to hemin-treated SS-*Tlr4^-/-^* mice, there was a trend toward decreased pro-fibrotic TGF-β expression in both liver and kidney samples (**Supplemental Figure 4**). Therefore one may speculate that long-term SS-*Tlr4^-/-^* mice may be protected from fibrosis.

In other I/R models, such as cardiac transplant, loss of endothelial TLR4/Trif-mediated signaling reduces neutrophil adhesion and recruitment (23). Our data is consistent with these models, as the livers of hemin-treated Townes SS-*Tlr4*^-/-^ mice demonstrate loss of *Ccl3* and *Cxcl2* gene upregulation, two chemokines important for the initiation of selectin-mediated rolling and leukocyte recruitment. Of note, in our kidney mRNA assessment, we did not see a significant change in *Ccl3* or *Cxcl2* mRNA changes; this likely reflects that kidneys lack cells similar to stellate cells and that renal disease in SCD is not characterized by inflammatory cell infiltrates. Last, our data demonstrates loss of heme-mediated release of Weibel-Palade bodies from the lung endothelium in SS *Tlr4^-/-^* mice. This is important as surface expression of von Willebrand factor and P-selectin are involved in platelet binding, leukocyte recruitment, and stasis (8, 28–31). Collectively, these data suggest that endothelial TLR4 response to heme leads to increased leukocyte recruitment, rolling, and adhesion to amplify I/R physiology.

With the advent of gene-therapy on the horizon for SCD, the importance of hematopoietic verses non-hematopoietic TLR4 signaling is a crucial distinction as TLR4 expression on hematopoietic cells is essential for bacterial clearance. As current transplant paradigms incorporate myelo-ablation, which increases risk of infections, strategies that reduce VOC but maintain effective pathogen clearance in SCD are desirable. Therefore, within the context of SCD, targeting of heme-mediated vascular TLR4 signaling, but not LPS-mediated TLR4 signaling, may be a strategy to prevent or decrease I/R injury while maintaining immune function.

One limitation of this work is we did not quantitate leukocyte recruitment during VOC physiology; however, we have previously demonstrated reduced leukocyte rolling in *Tlr4*^-/-^ mice transplanted with SS BM (8). Second, our studies have not evaluated SS-*Tlr4*^-/-^ mice for reductions in acute pain but recent studies by Lei et al. used BERK- SS-*Tlr4*^-/-^ mice to demonstrate a causal role of free heme in the genesis of acute and chronic sickle pain (12).

In conclusion, endothelial TLR4 signaling triggered by heme is critical for SCD VOC. We demonstrate the knockout of vascular, not hematopoietic, TLR4 signaling reduces heme-mediated inflammation and VOC. Overall, these data suggest that targeted inhibition of heme-mediated vascular endothelial TLR4 signaling may be a potential strategy to break the inflammatory cycle of I/R that is initiated by HbS-driven hemolysis.

## Data Availability Statement

The raw data supporting the conclusions of this article will be made available by the authors, without undue reservation.

## Ethics Statement

The animal study was reviewed and approved by University of Minnesota Institutional Animal Care and Use Committee.

## Author Contributions

JBec designed and performed experiments, analyzed results, and wrote the manuscript. FA performed experiments and analyzed results. CC performed experiments and analyzed results. RK performed experiments and analyzed results. DR-R performed experiments and analyzed results. ZK performed experiments and analyzed results. AN performed experiments and analyzed results. PZ performed experiments and analyzed results. JN performed experiments and analyzed results. RH designed experiments and reviewed manuscript. JBel designed experiments, analyzed results and wrote the manuscript. GV designed experiments, analyzed results, and wrote the manuscript. All authors contributed to the article and approved the submitted version.

## Funding

Research funding from NIH 5R01HL114567. ZK was supported by the Hematology Research Training Grant T32 L007062/HL/NHLBI. RK and DR-R received funding from University of Minnesota Life Sciences Undergraduate Research Program (LSSURP) 5R25HL088728-14.

## Conflict of Interest

JBec receives funding from Bayer not related to work herein. JBel and GV receive research funding from CSL Behring and Mitobridge (Astellas).

The remaining authors declare that the research was conducted in the absence of any commercial or financial relationships that could be construed as a potential conflict of interest.
