# Supplementary figures and images for: Endothelial TLR4 Expression Mediates Vaso-Occlusive Crisis in Sickle Cell Disease
Source: Front Immunol. 2021 Jan 19;11:613278. doi: 10.3389/fimmu.2020.613278 (PMC7851052; doi:10.3389/fimmu.2020.613278)

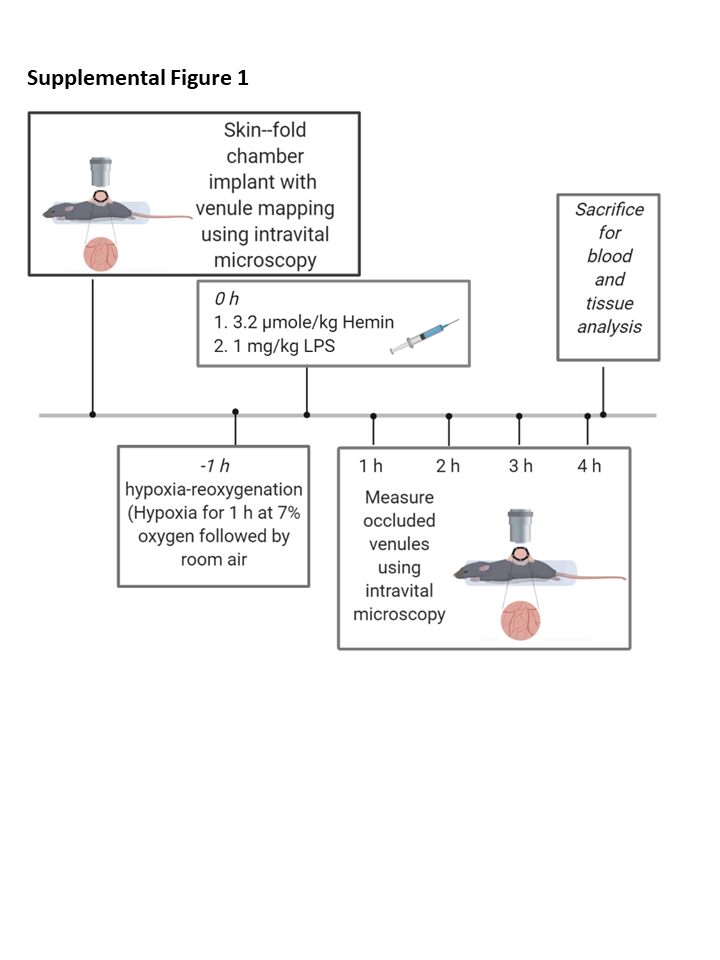

Supplement: Supplementary file 1 [file Image_1.tif]

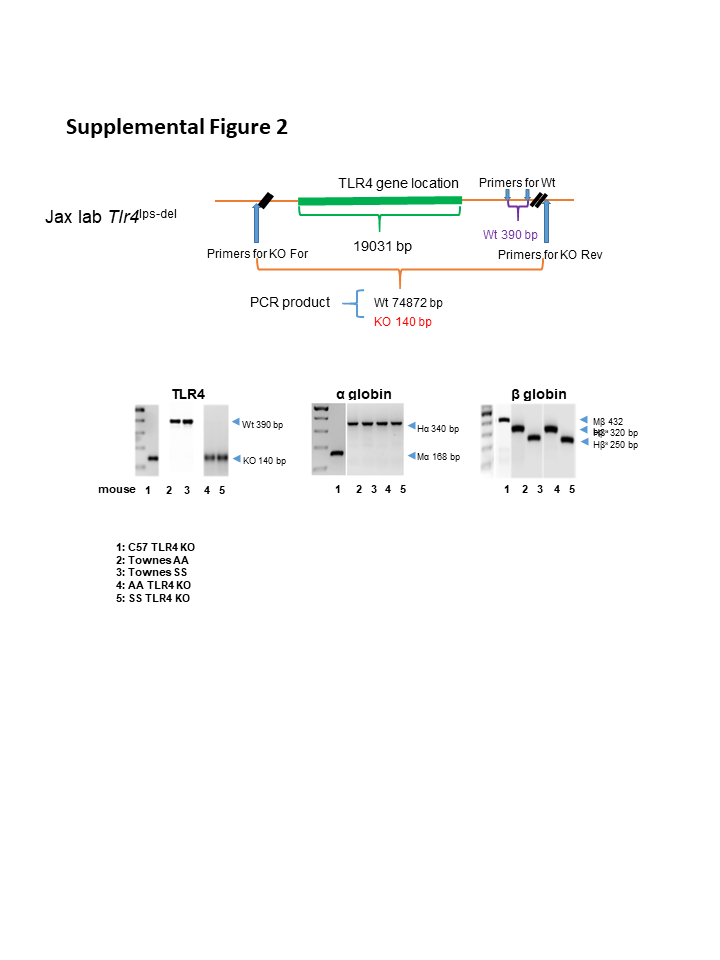

Supplement: Supplementary file 2 [file Image_2.tif]

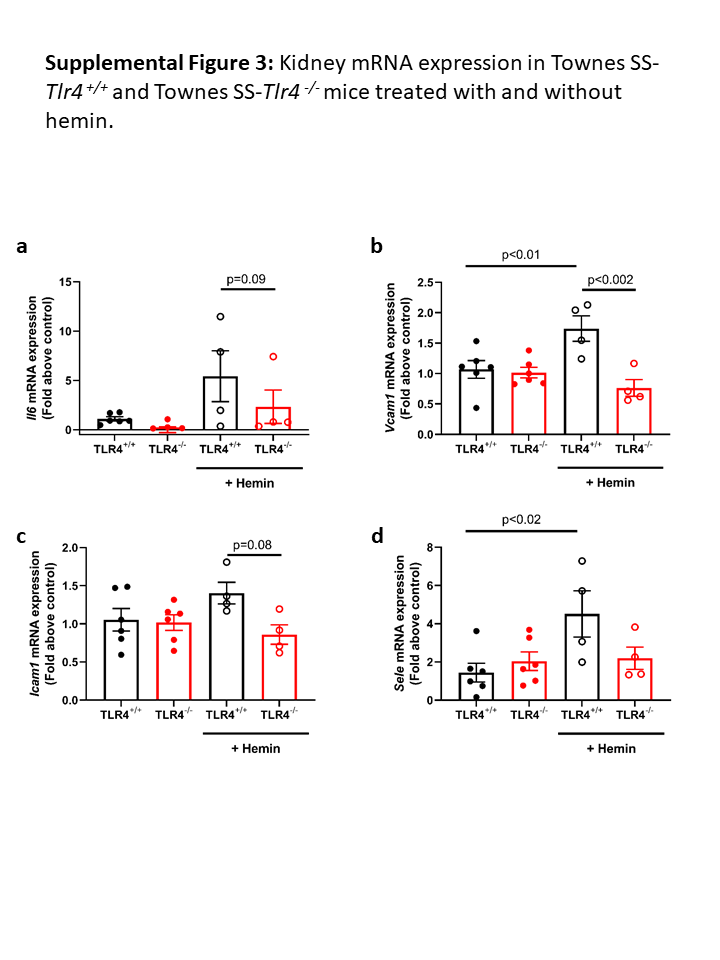

Supplement: Supplementary file 3 [file Image_3.tif]

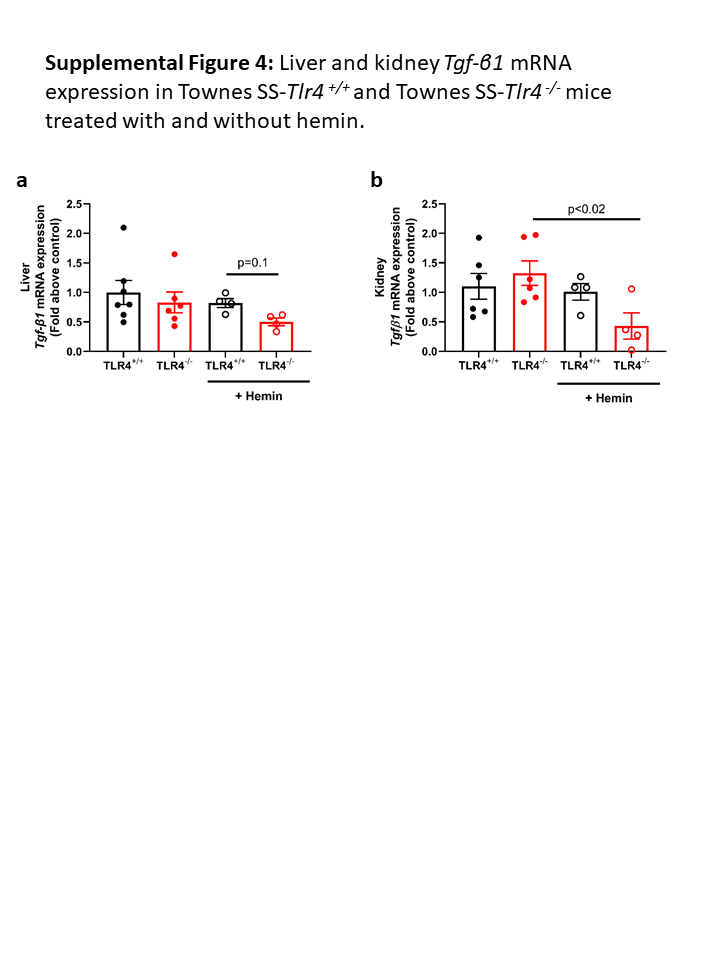

Supplement: Supplementary file 4 [file Image_4.tif]
